# Left Ventricular Dysfunction and CXCR3 Ligands in Hypertension: From Animal Experiments to a Population-Based Pilot Study

**DOI:** 10.1371/journal.pone.0141394

**Published:** 2015-10-27

**Authors:** Raffaele Altara, Yu-Mei Gu, Harry A. J. Struijker-Boudier, Lutgarde Thijs, Jan A. Staessen, W. Matthijs Blankesteijn

**Affiliations:** 1 Department of Pharmacology and Toxicology, Cardiovascular Research Institute Maastricht, Maastricht University, Maastricht, The Netherlands; 2 Studies Coordinating Centre, Research Unit Hypertension and Cardiovascular Epidemiology, KU Leuven Department of Cardiovascular Sciences, University of Leuven, Leuven, Belgium; 3 VitaK Research and Development, Maastricht University, Maastricht, The Netherlands; Instituto de Investigación Sanitaria INCLIVA, SPAIN

## Abstract

Detecting left ventricular (LV) dysfunction at an early stage is key in addressing the heart failure epidemic. In proteome profiling experiments in mice subjected either to aortic banding or sham, the circulating CXCR3 ligands monokine induced by interferon-γ (MIG) and interferon-γ inducible protein 10 (IP10) were 5 to 40 fold up-regulated at eight weeks. We assessed the diagnostic value of circulating NT-pro BNP and CXCR3 ligands (MIG, IP10, Interferon-inducible T-cell alpha chemo-attractant [I–TAC]) in patients with hypertension (≥140/90 mm Hg) associated with subclinical (*n* = 19) or symptomatic (*n* = 16) diastolic LV dysfunction on echocardiography and healthy controls. NT–pro BNP, MIG, IP10, I–TAC all increased (p ≤ 0.014) across the categories of worsening left ventricular dysfunction. In patients with symptomatic disease, MIG, IP10, and I–TAC increased 210% (p = 0.015), 140% (p = 0.007) and 120% (p = 0.035) more than NT-pro BNP. The optimal discrimination limits, obtained by maximizing Youden’s index were 246 pmol/L, 65 pg/mL, 93 pg/mL, and 24 pg/mL, respectively. The odds ratios associated with the four biomarkers were significant (p ≤ 0.010), ranging from 4.00 for IP10 to 9.69 for MIG. With adjustment for NT–pro BNP, the CXCR3 ligands retained significance (p ≤ 0.028). Adding optimized thresholds for the CXCR3 ligands to NT–pro BNP enhanced (p ≤ 0.014) the integrated discrimination improvement and the net reclassification improvement. In conclusion, congruent with the concept that inflammation plays a key role in the pathogenesis of LV dysfunction, MIG, IP10 and I–TAC add diagnostic accuracy over and beyond NT–pro BNP.

## Introduction

Heart failure is a major public health problem affecting more than more than 23 million patients worldwide [1]. Increased longevity [2] and the recently improved survival after first diagnosis [1] explain why the prevalence of heart failure will continue rising by 50% over the next 10–15 years. The associated disability entails high health care costs exceeding those of cancer [2] After the initial diagnosis, the estimated mortality rate is 25–28% at 1 year and 48–65% at 5 years [1]. Heart failure is a progressive condition, starting with the mere presence of risk factors, such as hypertension or diabetes (stage A) [3]. The next step includes asymptomatic changes in cardiac structure and function, as exemplified by left ventricular hypertrophy or impaired relaxation (stage B) [3]. Finally patients progress to clinically overt heart failure (stages C and D), disability, and death [3].

Screening tools that can identify heart failure patients early during the long asymptomatic course of the disease are of crucial importance in addressing the heart failure epidemic. Such tools not only allow timely intervention, but also help in characterizing the underlying molecular mechanisms and better targeting interventions. Circulating [4] or urinary [5] biomarkers reflecting inflammation [6],oxidative stress [7], cardiomyocyte injury [8], or left ventricular remodeling [5,9] are easily accessible and allow frequent re-evaluation. Inflammation is the driving force in causing myocardial damage in a wide variety of conditions, cardiac damage associated with myocardial ischemia, myocardial infarction, and viral myocarditis [10,11]. Our current study aimed to identify novel inflammatory biomarkers for left ventricular dysfunction by performing a screen of 40 inflammatory biomarkers, including CXCR3 ligands [12], in serum obtained from mice subjected to aortic banding. Next, we assessed biomarkers identified in the animal experiments as screening tools in a case-control study nested within the FLEmish study on Environment, Genes and Health Outcome (FLEMENGHO) [13,14].

## Methods

### Experimental studies in mice

The Animal Care and Use Committee of Maastricht University approved the experimental studies in male C57BL/6J mice (Harlan Laboratories, Horst, The Netherlands). As described in detail elsewhere [15], we induced pressure overload in by aortic banding. Control animals underwent the same surgical procedure without the actual tightening of the ligature (sham). We assessed left ventricular contractility at 4 or 8 weeks after the intervention under urethane anaesthesia (+dP/dt [16,17]), just before the animals (*n* = 30) were sacrificed by exsanguination through blood sampling from the abdominal aorta. It was then allowed to clot for 4 hours at 4°C before centrifugation at 2000 g for 10 minutes. Next, serum was removed, immediately aliquoted, and stored at –80°C. The profile of 40 inflammatory mediators was determined, using the Proteome Profiler Mouse Cytokine Array Kit, Panel A (R&D systems, Abingdon, United Kingdom) with improved fluorescence detection, as described previously. Membranes were incubated with 1 mL of pooled serum obtained from 4 to 6 animals per group. A total volume of 1000 μL (maximum possible volume) was applied to the membrane. To determine 40 extracellular cytokines in a semi-quantitative manner, we followed the manufacturer’s instructions available online at http://www.rndsystems.com/product_detail_objectname_ProteomeProfilerArray.aspx. We modified this protocol after step 9 to acquire semi-quantitative measurements by use of a fluorescence read-out [18].

### Nested case-control study

FLEMENGHO is a large-scale family-based study on the genetic epidemiology of cardiovascular phenotypes, for which recruitment started in 1985 and continued through 2008 [13,14]. The Ethics Committee of the University of Leuven approved the FLEMENGHO study. Participants gave written informed consent. As reported previously[5], we selected 35 cases and 35 controls from a cohort [19], who from 2005 until 2010 underwent echocardiography including tissue Doppler imaging by means of the Vivid7 Pro scanner (GE Vingmed, Horten, Norway) interfaced with a 2.5- to 3.5-MHz phased-array probe. We followed the recommendations of the American Society of Echocardiography [20] for data acquisition and the offline analysis. Cases were hypertensive patients with either asymptomatic (*n* = 19) or symptomatic (*n* = 16) diastolic left ventricular dysfunction and controls were normotensive healthy controls [5]. The diagnosis of left ventricular dysfunction relied on earlier published age-specific echocardiographic criteria for impaired relaxation and increased left ventricular filling pressure [5,19]. Participants reported cardiovascular, dyspnea, and respiratory symptoms by completing the London School of Hygiene questionnaires [21] and intake of medications and lifestyle characteristics via an observer-administered standardized questionnaire. Blood pressure was measured twice in the supine position, after echocardiography, using the validated [22] OMRON *705IT* device (OMRON Healthcare Europe BV, Nieuwegein, The Netherlands). Hypertension was a blood pressure of at least 140 mm Hg systolic or 90 mm Hg diastolic or use of antihypertensive drugs. Body mass index was weight in kilogram divided by height in meter squared.

With participants fasting for at least 6 hours, venous blood samples were drawn for measurement of serum cholesterol, plasma glucose, and biomarkers. Diabetes was identified by the use of antidiabetic drugs, a plasma glucose of at least 7.0 mmol/L, a self-reported diagnosis, or diabetes documented in practice or hospital records [23]. Blinded to the clinical data, we measured the levels of circulating inflammatory biomarkers by enzyme linked immunosorbent assay (ELISA), using the Human CXCL9/MIG Quantikine ELISA Kit, the Human CXCL10/IP10 Quantikine ELISA Kit and the Human CXCL11/I–TAC Quantikine ELISA (R&D Systems, Minneapolis MN). We also determined plasma N–terminal pro–atrial natriuretic peptide (NT–pro BNP) by a competitive enzyme immunoassay designed for research only (Biomedica Gruppe, Vienna, Austria) [24]. Standard NT–proBNP levels range 0 to 1000 pmol/L (median, 208 pmol/L; 95th, percentile 300 pmol/L) [24]. Because of insufficient plasma sample volume, the maximum number of cases and controls analyzed in our current manuscript amounted to 31 and 32, respectively; the minimum numbers were 29 cases and 29 controls.

We compared means by a *t*-test or ANOVA and proportions by Fisher’s exact test, using SAS software, version 9.3 (SAS Institute, Cary, NC, USA). Significance was a two-tailed

α–level of 0.05 or less. We normalized the distributions of the circulating biomarkers by logarithmic transformation. We modeled the odds of being a case in relation to the circulating biomarkers by logistic regression. We used mixed models to test the null hypothesis that increases in new biomarkers in patients with left ventricular dysfunction were identical to that observed for NT–pro BNP. We determined optimal discrimination limits for biomarkers by maximizing the Youden’s index (sensitivity plus specificity minus 1) [25]. We assessed the diagnostic accuracy of the inflammatory biomarkers, using the integrated discrimination improvement (IDI) and the net reclassification improvement (NRI) [26]. IDI is the difference between the discrimination slopes of basic models and basic models extended with a predictor variable. The discrimination slope is the difference in predicted probabilities (%) between cases and controls. NRI is the sum of the percentages of subjects reclassified correctly into cases and controls.

## Results

### Experimental studies in mice

We assessed cardiac hemodynamics in mice subjected to transverse aortic banding and in sham operated controls before and after dobutamine infusion. Baseline +dP/dt before infusion of dobutamine was similar in banded and control animals, averaging (SE) 129 ± 7 *vs*. 155 ± 3 mmHg/s at 4 weeks, and 149 ± 20 *vs*. 156 ± 13 mmHg/s at 8 weeks. However, upon maximal stimulation with dobutamine (6–7 ng/g/min), the increase in +dP/dt was attenuated (p < 0.05) in banded compared with control mice, averaging 239 ± 19 *vs*. 298 ± 17 mmHg/s at 4 weeks and 215 ± 19 *vs*. 296 ± 8 *vs*. mmHg/s at 8 weeks.

**Fig 1:**
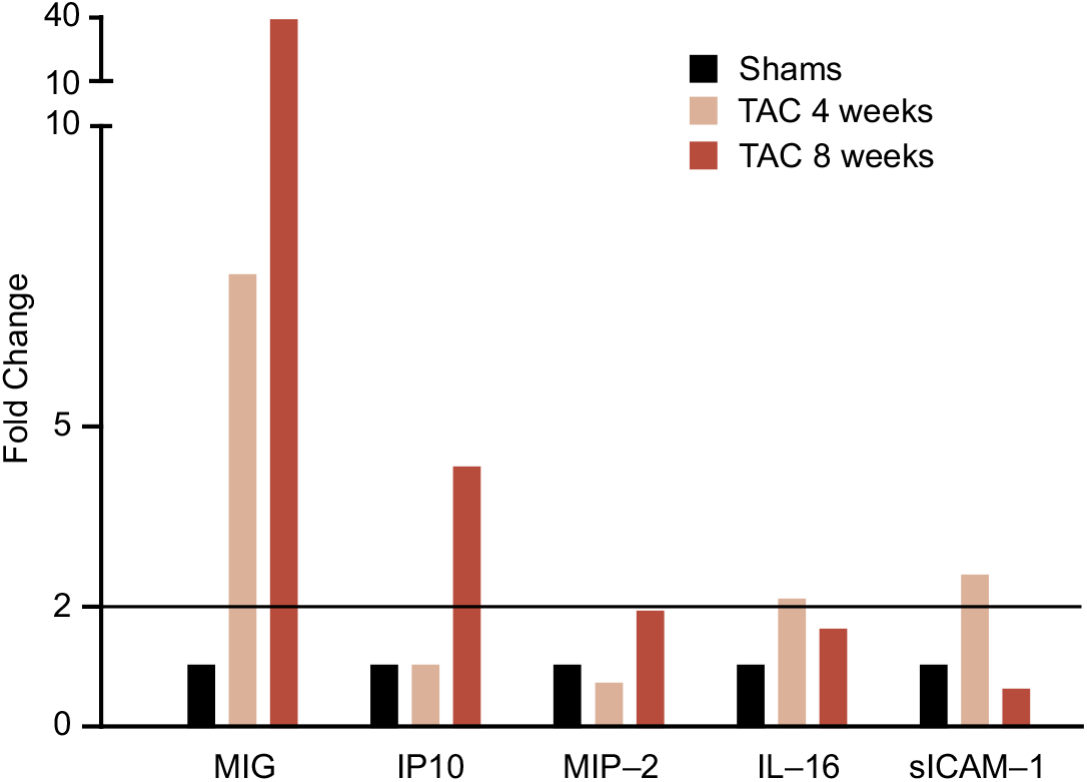
Expression profiles of the five most up-regulated cytokines in mice 4 and 8 weeks after aortic banding compared with sham operated animals. After transverse aortic constriction (TAC), expression profiles increased at least twice (dashed line). MIG, IP10, MIP–2, IL–16 and sICAM–1 indicate monokine induced by interferon–γ (CXCL9), interferon–γ inducible protein 10 (CXCL10), murine macrophage inflammatory protein–2, interleukin 16, and soluble intercellular adhesion molecule 1, respectively.

In the proteome profiling experiments, 5 of 40 cytokines stood out as being at least 2–fold up-regulated (Fig 1). They included in order of the degree of up-regulation: monokine induced by interferon–γ (MIG [CXCL9]), interferon–γ inducible protein 10 (IP10 [CXCL10]), murine macrophage inflammatory protein-2 (MIP–2), interleukin 16 (IL–16), and soluble intercellular adhesion molecule 1 (sICAM–1). MIG and IP-10 are ligands of the CXCR3 receptors, which are predominantly expressed by T-lymphocytes and natural killer cells [27]. CXCR3 receptors also bind Interferon-inducible T-cell alpha chemo-attractant (I–TAC [CXCL11]) [27]. Based on our current experiments in mice and the available literature [12,27], we carried MIG, IP10 and I-TAC further in the case-control study nested in the FLEMENGHO echocardiographic studies.

### Nested case-control study

Compared to healthy controls (Table 1), patients with subclinical left ventricular dysfunction, were older and had higher body mass index, blood pressure, and were more frequently on treatment with diuretics and β-blockers (p ≤ 0.029). Compared to patients with subclinical diastolic left ventricular dysfunction (Table 1), symptomatic patients were older, had higher plasma glucose, had a higher prevalence of coronary heart disease, and were more frequently on treatment with vasodilators (p ≤ 0.032). The echocardiographic measurements of the healthy controls and of the patients with subclinical or symptomatic left ventricular dysfunction appear in S1 Table.

**Table 1:** Clinical Characteristics of Controls and Cases.

Characteristics	Control (*n* = 32)	Subclinical LV dysfunction (*n* = 17)	Symptomatic LV dysfunction (*n* = 14)
**Number of subjects**			
** Women (%)**	14 (43.7)	9 (52.9)	8 (57.1)
** Smoker (%)**	11 (34.4)	4 (23.5)	2 (14.3)
** Drinker (%)**	28 (87.5)	12 (70.6)	7 (50.0)
** Coronary heart disease (%)**	1 (3.1)	1 (5.9)	6 (42.9)*
** Hypertensive drug treatment**	0	12 (70.6)‡	10 (71.4)
** Diuretics (%)**	0	7 (41.2)‡	7 (50.0)
** β-blockers (%)**	0	9 (52.9)‡	5 (35.7)
** RAS inhibitors (%)**	0	2 (11.8)	5 (35.7)
** Vasodilators (%)**	0	0	4 (28.6)*
**Mean characteristic (±SD)**			
** Age (year)**	62.8 ± 6.1	67.4 ± 7.6*	73.9 ± 8.3*
** Body mass index (kg/m^2^)**	25.0 ± 2.4	29.8 ± 4.3‡	30.2 ± 4.2
** Blood pressure**			
** Systolic (mm Hg)**	122.3 ± 9.0	150.9 ± 9.6‡	147.8 ± 21.4
** Diastolic (mm Hg)**	74.6 ± 4.9	81.7 ± 10.5*	77.8 ± 10.9
** Mean (mm Hg)**	90.5 ± 4.5	104.8 ± 8.6‡	101.1 ± 11.7
** Heart rate (beats per minute)**	62.5 ± 7.1	60.9 ± 8.8	64.6 ± 16.3
** Serum cholesterol (mmol/L)**	5.48 ± 0.75	5.60 ± 1.08	5.41 ± 1.19
** Blood glucose (mmol/L)**	4.90 ± 0.39	4.74 ± 0.54	5.41 ± 0.70†

LV indicates left ventricle. Mean arterial pressure was diastolic blood pressure plus one third of the difference between systolic and diastolic blood pressure. Inhibitors of the renin-angiotensin system included angiotensin-converting enzyme inhibitors and angiotensin type–1 receptor blockers. Vasodilators were calcium channel blockers and α–blockers. P–values for trend across categories were significant (p ≤ 0.025) except for the proportion of women and smokers and the mean values of heart rate and serum cholesterol (p ≥ 0.39). Significance of the difference with left adjacent group:

* p ≤ 0.05

† p ≤ 0.01; and

‡ p ≤ 0.001.

Circulating levels of MIG, IP10, I–TAC and NT–pro BNP all increased (p ≤ 0.014) across the categories of worsening left ventricular dysfunction (Table 2). Compared with healthy controls, circulating levels of the four biomarkers were similar in patients with subclinical left ventricular dysfunction (p ≥ 0.14), whereas they were significantly elevated (p ≤ 0.011) in symptomatic patients (Table 2). In patients with symptomatic disease, MIG, IP10, and

**Table 2:** Circulating CXCR3 Ligands and NT–pro BNP in Controls and Cases.

Biomarker	Characteristics	Control	Subclinical LV dysfunction	Symptomatic LV dysfunction	p for trend
**Monokine induced by interferon–**γ **(MIG [CXCL9])**	*n*° of participants	32	17	14	
	Level (pg/mL)	40.3 (29.9–54.2)	56.7 (28.6–98.0)	111.0 (39.6–332.5) ‡	0.014
**Interferon–**γ **inducible protein 10 (IP10 [CXCL10])**	*n*° of participants	32	17	14	
	Level (pg/mL)	80.7 (55.0–113.7)	105.1 (63.4–140.5)	148.2 (81.4–213.2)*	0.001
**Interferon-inducible T-cell alpha chemo-attractant (I–TAC [CXCL11]**	*n*° of participants	31	13	13	
	Level (pg/mL)	14.9 (7.80–22.4)	22.4 (7.80–35.2)	32.3 (23.2–56.9)†	0.008
**N–terminal pro–brain natriuretic peptide (NT–pro BNP)**	*n*° of participants	29	16	13	
	Level (pmol/L)	202.1 (150.6–305.9)	284.3 (156.0–544.6)	380.7 (258.8–663.4)†	0.005

LV indicates left ventricle. Biomarker levels are geometric means (interquartile ranges).

Significance with healthy controls:

* p ≤ 0.05

† p ≤ 0.01; and

‡ p ≤ 0.001.

**Fig 2:**
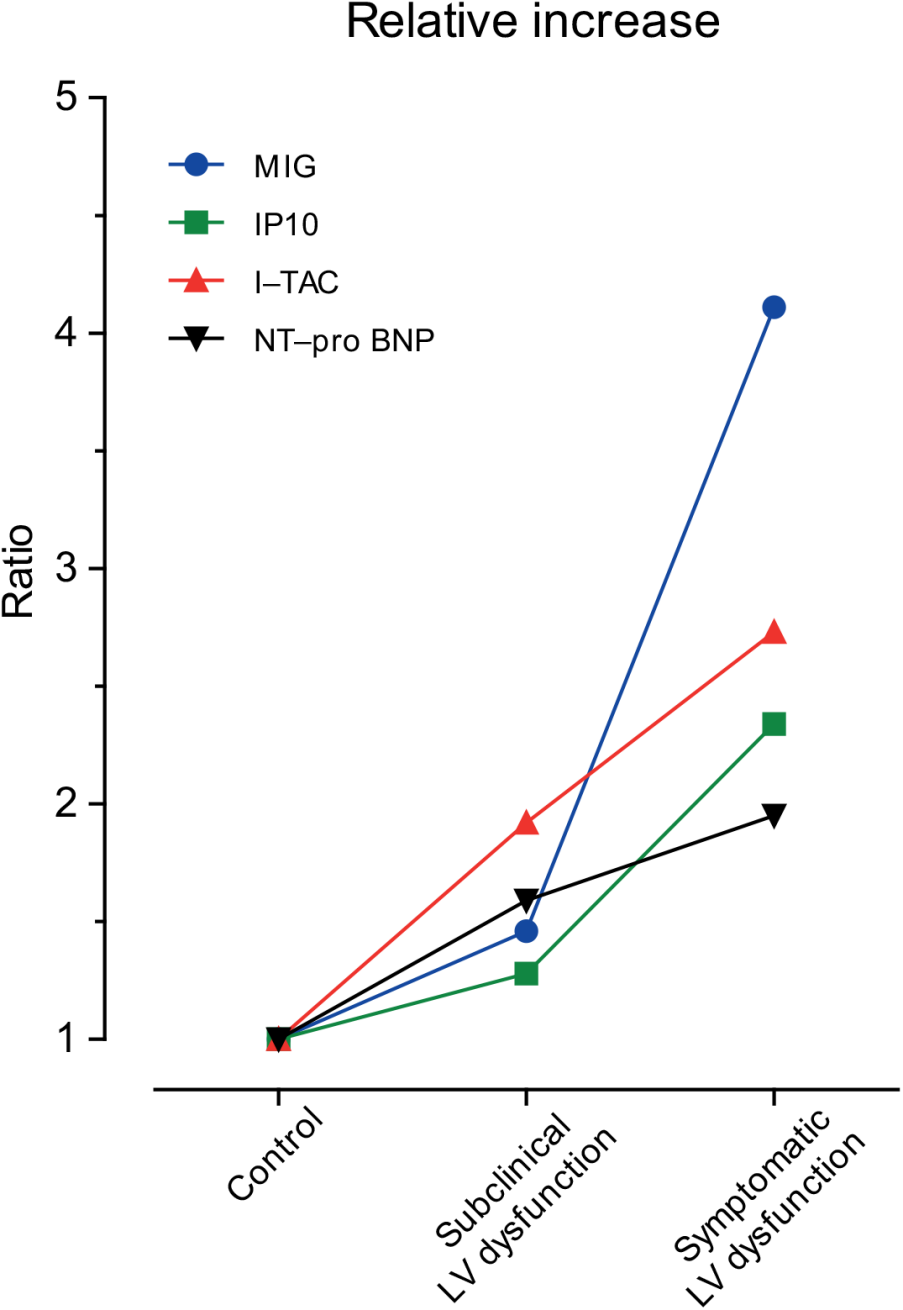
Circulating biomarkers in patients with subclinical and symptomatic left ventricular dysfunction, normalized to each marker’s mean in healthy controls. The vertical axis therefore expresses the average ratio in patients relative to the mean in all healthy controls combined.

I–TAC increased 210% (p = 0.015), 140% (p = 0.007) and 120% (p = 0.035) more than NT–pro BNP (Fig 2). By maximizing Youden’s index, the optimal discrimination limits of MIG, IP10, I–TAC and NT–pro BNP respectively were 65 g/mL, 93 pg/mL, 24 pg/mL, and 246 pmol/L (S2 Table). Table 3 shows the odds ratios for having left ventricular dysfunction in relation to the biomarkers. In unadjusted analyses, the odds ratios associated with the four biomarkers were all significant (p ≤ 0.010), ranging from 4.00 for IP10 to 9.69 for MIG. With adjustment for NT–pro BNP, the CXCR3 ligands retained significance (p ≤ 0.028). The odds ratios were 6.95 for MIG, 3.74 for IP10 and 12.3 for I–TAC. Adding the optimized thresholds for the CXCR3 ligands to basic models including NT–pro BNP alone or in combination with age and body mass index (Table 4) significantly improved IDI (p ≤ 0.038) and NRI (p ≤ 0.005) except for IP10 in models also including age and body mass index (p = 0.42). Sensitivity analyses, in which we computed odds ratios (S3 Table), IDI and NRI (S4 Table), with the biomarkers expressed on a continuous scale, were largely confirmatory, although for IDI significance was only reached for the three CXCR3 ligands combined added to a basic model only including NT–pro BNP.

**Table 3:** Odds Ratios Expressing the Risk of Left ventricular Dysfunction in Relation to Optimized Biomarker Thresholds.

Biomarker	Unadjusted odds ratio	p	Odds ratio adjusted for NT–pro BNP	p
**MIG (≥65 pg/mL)**	9.69 (2.73 to 34.4)	0.0004	6.95 (1.81 to 26.6)	0.005
**IP10 (≥93 pg/mL)**	4.00 (1.40 to 11.4)	0.010	3.74 (1.15 to 12.1)	0.028
**I–TAC (≥24 pg/mL)**	7.09 (2.07 to 24.3)	0.002	12.3 (2.40 to 63.3)	0.003
**NT–pro BNP (≥246 pmol/L)**	6.98 (2.19 to 22.2)	0.001	…	…

Abbreviations of the biomarkers are spelled out in Table 2. Odds ratios (95% confidence intervals) express the risk associated with a biomarker concentration above the optimized threshold.

**Table 4:** Net Reclassification Improvement and Integrated Discrimination Improvement by Adding Optimized Thresholds of the CXCR3 Ligands to the Basic Models.

Variables in basic models biomarkers	Integrated discrimination improvement		Net reclassification improvement	
	Δ% (95% confidence interval)	p	Δ% (95% confidence interval)	p
**NT-pro BNP**				
** MIG**	12.9 (4.01 to 21.8)	0.004	89.7 (45.9 to 133.4)	<0.0001
** IP10**	7.51 (1.55 to 13.5)	0.014	69.0 (20.7 to 117.3)	0.005
** I–TAC**	17.8 (7.67 to 28.0)	0.006	84.3 (36.5 to 132.0)	0.0005
** MIG + IP10 + I–TAC**	22.8 (10.4 to 35.2)	0.0003	124.2 (82.0 to 166.6)	<0.0001
**NT-pro BNP, age and body mass index**				
** MIG**	5.45 (0.29 to 10.6)	0.038	110.3 (68.3 to 152.4)	<0.0001
** IP10**	0.63 (–0.90 to 2.16)	0.42	69.0 (20.7 to 117.3)	0.005
** I–TAC**	9.64 (1.33 to 18.0)	0.023	154.6 (120.3 to 188.8)	<0.0001
** MIG + IP10 + I–TAC**	18.8 (9.62 to 28.0)	<0.0001	192.9 (179.1 to 206.6)	<0.0001

Abbreviations of the biomarkers are spelled out in Table 2. The net reclassification improvement is the sum of the percentages of subjects reclassified correctly in cases and controls. The integrated discrimination improvement is the difference between the discrimination slopes of the extended and basic models. The discrimination slope is the difference in predicted probabilities between cases and controls. Cases were patients with subclinical or symptomatic diastolic left ventricular dysfunction. Controls were healthy people.

## Discussion

The main finding of our study was that by performing an unbiased screen for inflammatory biomarkers in a mouse model of heart failure, we identified a cluster of three related chemokines that were present in elevated concentrations in the plasma of patients with symptomatic diastolic left ventricular dysfunction. These three CXCR3 ligands enhanced diagnostic accuracy over and beyond NT–pro BNP, as exemplified by the integrated discrimination improvement and the net reclassification improvement.

Heart failure is a major and growing public health problem with a complex etiology [28]. The diagnosis of heart failure, particularly in the early stage when clinical symptoms are still mild of even absent, remains a challenge. Several circulating biomarkers are currently considered in the diagnosis and management of heart failure. BNP and NT–proBNP are natriuretic peptides, which are released from the myocardium upon excessive stretch. Their circulating levels are elevated in heart failure patients and useful for confirmation of the diagnosis in patients with dyspnea [3]. Troponin T and I are markers of myocardial damage when cardiac ischemia or myocardial infarction occurs. After the introduction of a high-sensitive assay, the application of troponin T and I extended to myocardial injury as a consequence of advanced heart failure [4]. Wang and colleagues measured 10 biomarkers, including B–type natriuretic peptide (BNP) and NT–pro BNP in 3209 participants attending a routine examination cycle of the Framingham Heart Study [29]. During a median follow-up of 7.4 year, 207 participants died and 169 had a first major cardiovascular event. In Cox proportional-hazards models with adjustments applied for conventional risk factors, BNP, but not NT–pro BNP was a strong predictor. The Framingham investigators also combined circulating biomarkers into multimarker scores [4]. However, the addition of multimarker scores to conventional risk factors resulted in only small enhancements in risk stratification. As reviewed elsewhere [30], these Framingham findings [4] exemplify the need to develop new biomarkers with higher diagnostic and predictive value when used alone or in combination.

In the past decade, the research community made large efforts to identify novel biomarkers that would allow a better characterization of the disease process underlying left ventricular dysfunction and an improved stratification of prognosis and success of pharmacotherapy. C-reactive protein is an independent predictor of adverse outcomes in patients with acute or chronic heart failure [28]. Other biomarkers that established a link between heart failure and inflammation include tumor necrosis factor-alpha [31], and the pro-inflammatory (e.g. interleukin–6 [32]) and anti-inflammatory (interleukin–10 [33]) cytokines.

Inflammatory biomarkers probably contain valuable information with respect to the molecular mechanisms initiating left ventricular dysfunction and its progression to symptomatic heart failure. In the present study, we focused on the circulating CXCR3 ligands, MIG, IP10 and I–TAC. These CXCR3-agonistic cytokines are involved in autoimmune diseases, such as rheumatoid arthritis and systemic lupus erythematosus [12]. They play a role in recruiting immune cells to the sites of inflammation. Hypertensive patients also have elevated levels of circulating MIG, IP10 and I–TAC [34–37]. Whether this is causal or just reflecting target organ damage remains currently unknown. In this context it should be noted that in our patients with left ventricular dysfunction, the average blood pressure was elevated, while the majority were receiving antihypertensive drugs. However, all CXCR3 ligands showed higher average levels in patients with symptomatic left ventricular dysfunction, despite the fact that the blood pressure tended to be lower in these patients. These observations suggest that the elevated levels of MIG and I–TAC are not solely the result of an increased blood pressure, but also correlate with the degree of left ventricular dysfunction.

An important prerequisite for a biomarker is its involvement in the disease. Is it just an innocent bystander or a causal component of the pathological process [4]? Inflammatory mediators drive cardiac remodeling in response to ischemia, myocarditis and grafting [38,39]. However, hypertension was probably the trigger of left ventricular dysfunction in our participants. Although hemodynamic stress can induce an inflammatory state in the myocardium, the inflammatory triggers are not entirely certain [10]. The CXCR3 ligands MIG, IP10 and I-TAC are involved in the recruitment of immune cells, particularly T-lymphocytes, into sites of inflammation [12,40]. Augmented infiltration of CD4+ T-lymphocytes is associated with a rapid deterioration of the ejection function in a rat model of spontaneous hypertension [41]. Although the precise roles of the different T-lymphocyte subsets in the inflammatory response to cardiac hemodynamic stress have not yet been fully clarified, our current data support the involvement of CXCR3 ligands in left ventricular dysfunction.

One of the limitations of the present study is that due to the experimental setup we could not identify the source of the CXCR3 ligands that were elevated in the circulation of patients with left ventricular dysfunction. Inducible expression of IP10 has been demonstrated in monocytes, endothelial cells and fibroblasts [42,43] and regulates the reparative response in an experimental model of myocardial infarction [44] To our knowledge, no previous study reported on the functional effects of CXCR3 agonists on the response of the heart to hemodynamic overload. Additional work in animal models will be needed to resolve this question. Other issues that must be addressed after this pilot study, is independent confirmation of the diagnostic accuracy of circulating CXCR3 ligands in patients with early stages of left ventricular dysfunction and validation of these new markers in terms of outcome and responses to treatment.

In conclusion, left ventricular dysfunction is associated with elevated circulating levels of the CXCR3 ligands MIG, IP10 and I–TAC. Adding the levels of these chemokines to the risk prediction model provided a significant net reclassification and integrated discrimination improvement. However, further experimental, clinical and epidemiological studies are required to substantiate the findings from our pilot study.

## Supporting Information

S1 Table Echocardiographic characteristics in cases and controls. (DOCX)

S2 Table Optimized diagnostic thresholds for the circulating biomarkers in relation to left ventricular dysfunction. (DOCX)

S3 Table Odds ratios expressing the risk of left ventricular dysfunction in relation to biomarkers analyzed as continuous variables. (DOCX)

S4 Table Net reclassification improvement and integrated discrimination improvement by adding CXCR3 ligands as continuous variables to basic models. (DOCX)
