# Supplementary material for: Left Ventricular Dysfunction and CXCR3 Ligands in Hypertension: From Animal Experiments to a Population-Based Pilot Study
Source: PLoS One. 2015 Oct 27;10(10):e0141394. doi: 10.1371/journal.pone.0141394 (PMC4624781; doi:10.1371/journal.pone.0141394)
Supplement: S1 Table — (DOCX) [file pone.0141394.s001.docx]

**S1 Table**

**Echocardiographic Characteristics in Cases and Controls**

| **Characteristic** | **Control (*n* = 32)** | **Subclinical  LV dysfunction  (*n* = 17)** | **Symptomatic   LV dysfunction  (*n* = 14)** |
| --- | --- | --- | --- |
| **Conventional echocardiography** |  |  |  |
| **Left atrial diameter (cm)** | 3.92 ± 0.47 | 4.38 ± 0.47† | 4.49 ± 0.65 |
| **LV internal diameter (cm)** | 4.95 ± 0.47 | 4.98 ± 0.51 | 5.38 ± 0.75 |
| **Interventricular septum (cm)** | 0.98 ± 0.14 | 1.19 ± 0.21‡ | 1.08 ± 0.17 |
| **Posterior wall (cm)** | 0.91 ± 0.12 | 1.08 ± 0.16‡ | 0.99 ± 0.14 |
| **LV mass index (g/m2)** | 92.8 ± 16.5 | 119.0 ± 27.0† | 110.0 ± 16.6 |
| **LV hypertrophy (%)** | 0 | 41.2‡ | 28.6 |
| **Ejection fraction (%)** | 70.7 ± 6.2 | 70.8 ± 9.5 | 61.0 ± 14.6 |
| **Ejection fraction<50% (%)** | 6.3 | 11.8 | 50.0* |
| **Transmitral Doppler data** |  |  |  |
| **E peak (cm/s)** | 71.3 ± 10.7 | 73.8 ± 16.2 | 68.6 ± 24.4 |
| **A peak (cm/s)** | 67.7 ± 11.0 | 89.3 ± 16.0‡ | 84.4 ± 15.8 |
| **E/A ratio** | 1.08 ± 0.23 | 0.85 ± 0.26† | 0.82 ± 0.25 |
| **Tissue Doppler velocities** |  |  |  |
| **e’ peak (cm/s)** | 10.6 ± 1.97 | 6.60 ± 1.83‡ | 7.55 ± 1.83 |
| **a’ peak (cm/s)** | 11.4 ± 1.86 | 10.8 ± 1.96 | 10.4 ± 2.64 |
| **e’/a’ ratio** | 0.97 ± 0.30 | 0.64 ± 0.24‡ | 0.76 ± 0.22 |
| **E/e’ ratio** | 6.87 ± 1.08 | 11.5 ± 2.27‡ | 9.27 ± 3.33* |

LV indicates left ventricle. P‑values for trend across categories were significant (p ≤ 0.022) except for LV internal diameter E peak, and a’ peak (p ≥ 0.16). Significance of the difference with the left adjacent group: * p ≤ 0.05; † p ≤ 0.01; and ‡ p ≤ 0.001.
