# Supplementary material for: Left Ventricular Dysfunction and CXCR3 Ligands in Hypertension: From Animal Experiments to a Population-Based Pilot Study
Source: PLoS One. 2015 Oct 27;10(10):e0141394. doi: 10.1371/journal.pone.0141394 (PMC4624781; doi:10.1371/journal.pone.0141394)
Supplement: S2 Table — (DOCX) [file pone.0141394.s002.docx]

**S2 Table**

**Optimized Diagnostic Thresholds for the Circulating Biomarkers in Relation to Left Ventricular Dysfunction**

| **Biomarkers** | **Optimal  discrimination  limit** | **Sensitivity** | **Specificity** | **Positive  predictive  value** | **Negative  predictive  value** | **Misclassification  rate** |
| --- | --- | --- | --- | --- | --- | --- |
| **MIG (pg/mL)** | 65 | 0.58 | 0.88 | 0.82 | 0.68 | 0.27 |
| **IP10 (pg/mL)** | 93 | 0.65 | 0.69 | 0.67 | 0.67 | 0.33 |
| **I–TAC (pg/mL)** | 24 | 0.58 | 0.84 | 0.75 | 0.70 | 0.25 |
| **NT–pro BNP (pmol/L)** | 246 | 0.76 | 0.69 | 0.71 | 0.74 | 0.25 |

Abbreviations of the biomarkers are spelled out in Table 2. The optimal discrimination limits were obtained by maximizing the Youden’s index (the maximum of sensitivity plus specificity minus 1).
