# Supplementary material for: Left Ventricular Dysfunction and CXCR3 Ligands in Hypertension: From Animal Experiments to a Population-Based Pilot Study
Source: PLoS One. 2015 Oct 27;10(10):e0141394. doi: 10.1371/journal.pone.0141394 (PMC4624781; doi:10.1371/journal.pone.0141394)
Supplement: S3 Table — (DOCX) [file pone.0141394.s003.docx]

**S3 Table**

**Odds Ratios Expressing the Risk of Left ventricular Dysfunction in Relation to Biomarkers Analyzed as Continuous Variables**

| **Biomarkers** | **Unadjusted  odds ratio** | **p** | **Odds ratio  adjusted for NT-pro BNP** | **p** |
| --- | --- | --- | --- | --- |
| **MIG** | 2.03 (1.20 to 3.43) | 0.008 | 1.68 (0.98 to 2.87) | 0.057 |
| **IP10** | 2.12 (1.13 to 3.97) | 0.019 | 1.91 (0.95 to 3.84) | 0.069 |
| **I–TAC** | 2.13 (1.21 to 3.75) | 0.009 | 2.77 (1.27 to 6.02) | 0.010 |
| **NT–pro BNP** | 2.72 (1.34 to 5.54) | 0.006 | … | … |

Abbreviations of the biomarkers are spelled out in Table 2. Odds ratios (95% confidence intervals) express the risk associated with a doubling of the biomarker.
