# Supplementary material for: Left Ventricular Dysfunction and CXCR3 Ligands in Hypertension: From Animal Experiments to a Population-Based Pilot Study
Source: PLoS One. 2015 Oct 27;10(10):e0141394. doi: 10.1371/journal.pone.0141394 (PMC4624781; doi:10.1371/journal.pone.0141394)
Supplement: S4 Table — (DOCX) [file pone.0141394.s004.docx]

**S4 Table**

**Net Reclassification Improvement and Integrated Discrimination Improvement by Adding CXCR3 Ligands as Continuous Variables to Basic Models**

| **Variables in basic models  biomarkers** | **Integrated discrimination improvement** | |  | **Net reclassification improvement** | |
| --- | --- | --- | --- | --- | --- |
|  | **Δ%  (95% confidence interval)** | **p** |  | **Δ%  (95% confidence interval)** | **p** |
| **NT–pro BNP** |  |  |  |  |  |
| **MIG** | 6.15 (–0.38 to 12.7) | 0.065 |  | 55.5 (5.98 to 104.4) | 0.028 |
| **IP10** | 5.45 (–0.43 to 11.3) | 0.38 |  | 41.4 (–8.70 to 91.5) | 0.11 |
| **I–TAC** | 12.9 (3.33 to 22.5) | 0.008 |  | 70.9 (20.5 to 121.2) | 0.006 |
| **All biomarkers** | 12.5 (2.63 to 22.3) | 0.013 |  | 63.7 (12.6 to 114.8) | 0.015 |
| **NT–pro BNP,  age and body mass index** |  |  |  |  |  |
| **MIG** | 1.89 (–1.28 to 5.06) | 0.24 |  | 96.6 (51.5 to 141.6) | <0.0001 |
| **IP10** | –0.02 (–0.21 to 0.18) | 0.85 |  | 6.90 (–44.4 to 58.2) | 0.79 |
| **I–TAC** | 3.72 (–1.39 to 8.84) | 0.15 |  | 115.4 (71.7 to 159.2) | <0.0001 |
| **All biomarkers** | 4.73 (–0.72 to 10.2) | 0.089 |  | 130.6 (90.0 to 171.1) | <0.0001 |

Abbreviations of the biomarkers are spelled out in Table 2. The net reclassification improvement (NRI) is the sum of the percentages of subjects reclassified correctly in cases and controls. The integrated discrimination improvement (IDI) is the difference between the discrimination slopes of the extended and basic models. The discrimination slope is the difference in predicted probabilities (%) between cases and controls. Cases were patients with subclinical or symptomatic diastolic left ventricular dysfunction. Controls were healthy people.
